# Nuclear Receptors Regulate Intestinal Inflammation in the Context of IBD

**DOI:** 10.3389/fimmu.2019.01070

**Published:** 2019-05-14

**Authors:** Victoria Klepsch, Alexander R. Moschen, Herbert Tilg, Gottfried Baier, Natascha Hermann-Kleiter

**Affiliations:** ^1^Translational Cell Genetics, Department of Pharmacology and Genetics, Medical University of Innsbruck, Innsbruck, Austria; ^2^Department of Internal Medicine I, Gastroenterology, Endocrinology and Metabolism, Medical University of Innsbruck, Innsbruck, Austria

**Keywords:** nuclear receptor, intestinal barrier homeostasis, immune system, microbiota, inflammatory bowel disease

## Abstract

Gastrointestinal (GI) homeostasis is strongly dependent on nuclear receptor (NR) functions. They play a variety of roles ranging from nutrient uptake, sensing of microbial metabolites, regulation of epithelial intestinal cell integrity to shaping of the intestinal immune cell repertoire. Several NRs are associated with GI pathologies; therefore, systematic analysis of NR biology, the underlying molecular mechanisms, and regulation of target genes can be expected to help greatly in uncovering the course of GI diseases. Recently, an increasing number of NRs has been validated as potential drug targets for therapeutic intervention in patients with inflammatory bowel disease (IBD). Besides the classical glucocorticoids, especially PPARγ, VDR, or PXR-selective ligands are currently being tested with promising results in clinical IBD trials. Also, several pre-clinical animal studies are being performed with NRs. This review focuses on the complex biology of NRs and their context-dependent anti- or pro-inflammatory activities in the regulation of gastrointestinal barrier with special attention to NRs already pharmacologically targeted in clinic and pre-clinical IBD treatment regimens.

## Introduction

**Figure 1:**
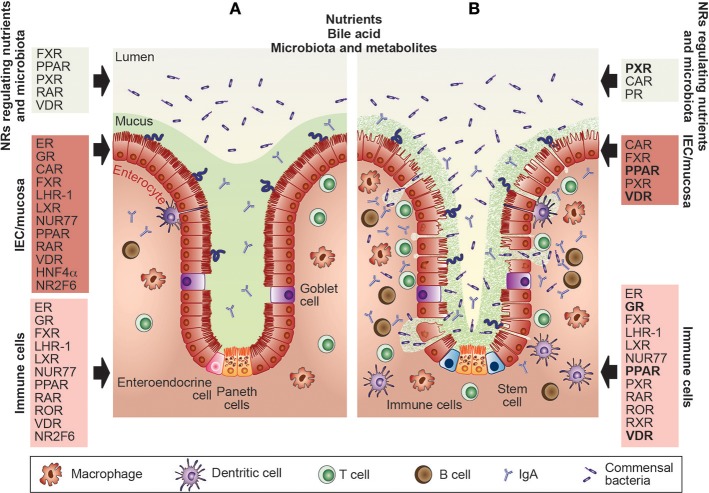
Nuclear receptors are essential for the maintenance of gut homeostasis and have already been targeted in IBD patients. **(A)** Within the healthy gastrointestinal system, nuclear receptors (NRs) such as FXR, PPAR, PXR, RAR, or VDR are well-known sensors of nutrients, toxic dietary products, specific host-bacterial metabolites, and bile acid. Intestinal barrier function and epithelial intestinal cell integrity are dependent on the appropriate function of the ER, GR, CAR, FXR, LHR-1, LXR, NUR77, PPAR, RAR, VDR, HNF4α, and NR2F6 which regulate mucus secretion, expression of tight junction proteins autophagy, circadian clock as well as goblet and paneth cell numbers. Also, NRs such as the ER, GR, FXR, LHR-1, LXR, NUR77, PPAR, RAR, ROR, VDR HNF4α, and NR2F6 contribute to gut homeostasis by shaping intestinal immune cell development, and the composition and effector functions of macrophages, dendritic cells, T and B cells. **(B)** The primary protective role of the NRs in the pathophysiology of inflammatory bowel diseases has been validated in pre-clinical animal models and clinical trials. NRs targeted by therapeutic drugs in IBD patients are GR, PPAR, PXR, and the VDR (highlighted in bold), NRs tested in preclinical mouse models are CAR, ER, FXR, LHR-1, LXR, NUR77, PPAR, PXR, RAR, ROR, and RXR; thus, novel concepts integrating NR, and gastrointestinal physiology have been integrated into the development of effective therapies. CAR, constitutive androstane receptor; ER, estrogen receptor; FXR, farnesoid X receptor; GR, glucocorticoid receptor; HNF4α, hepatocyte nuclear factor-4-alpha; IBD, inflammatory bowel disease; IECs, intestinal epithelial cells; LRH, liver-related homolog; LXR, liver X receptor; NR2F6, nuclear receptor subfamily 2 group F member 6; NR4A1/2, nuclear receptor subfamily 4 group A member 1/2 (NUR77, NUR1); PPAR, peroxisome proliferator-activated receptor; PXR, pregnane X receptor; RAR, retinoic acid receptor; RevErb, nuclear receptor subfamily 1, group D, member 1; ROR, RAR-related orphan receptor; RXR, retinoid X receptor; VDR, vitamin D receptor.

This review is based on the most recent advances in our understanding of the complex biology of nuclear receptors (NRs) within both the healthy and inflamed intestinal tract (Tables 1–3) and the emerging number of ligands successfully used in preclinical and clinical trials (Table 4) to target inflammation and treat inflammatory bowel disease (IBD) without focusing on intestinal infections (1–5). In general, NRs enable fine-tuning of cellular processes to environmental changes such as external milieu signals and the cell-intrinsic metabolic state and are therefore ideally suited as targets for therapeutic interventions (6–9). Within the gastrointestinal (GI) system, NRs are highly expressed and well-known sensors of nutrients, hormones, and specific host-bacterial metabolites (1–5). Gut physiology is regulated by several nuclear receptors such as ERβ (NR3B2), GR (NR3C1), FXR (NR1H), PPARγ (NR1C3), PXR (NR1I2), RARα (NR1B1), VDR (NR1I1), HNF4α (NR2A1), or NR2F6 (Ear2) which have been demonstrated to play fundamental roles in epithelial intestinal cell integrity and especially in shaping intestinal immune cell composition and function (Figure 1) (9–14).

**Table 1:** Steroidal nuclear receptors.

**Nuclear receptor**	**Spec**	**Model**	**Study outcome**	**References**
ER α/β NR3A1/2 (protective)	hu	Biopsies	*ERB* mRNA is decreased in IBD patients	(1, 2)
		Blood	Altered ERα expression (increased) and ERβ (decreased) in T lymphocytes from IBD patients	(3)
	mo	Spontaneous	Altered epithelial barrier in KO mice, decreased *Erb* mRNA levels	(2, 4)
		AOM/DSS	*ERβ^−/−^* mice are more susceptible to clinical AOM/DSS colitis-associated colorectal cancer	(5)
			Estrogens promote colon cancer development by impairing the mucosal responses	(6)
		Chemical	*ERb* expression in female mice protected against DSS colitis (male mice are not protected)	(7)
GR NR3C1 (controversial)	hu	Biopsies	IBD patients without steroid treatment showed increased *GR* expression	(8)
			Enhanced *GR* mRNA levels in leukocytes of UC patients	(9)
			*hGRb* mRNA expression in PBMCs is a novel predictor of glucocorticoid response in UC patients	(10)
			*hGRb* mRNA expression was significantly enhanced in active stage of UC	(11)
			UC patients are positive for *GRa* and *GRb* expression; *GRb* expression negatively correlates with GC response in UC patients	(12)
			IBD may be associated with GR polymorphisms	(13)
			GR isoform expression does not predict steroid treatment response in IBD patients	(14)
			GR levels increase in UC patients responding to GCS therapy	(15)
			GRβ^+^ cells are increased in GC-resistant group than control and GC-sensitive group	(16)
			No significant associations between GR gene polymorphisms and GR resistance in IBD treatment	(17)
			GR expression was downregulated in IBD patients	(18)
			No difference in GR expression in patients vs. healthy controls could be detected	(19)
	mo	Chemical	GR in myeloid cells essential to achieve resolution of DSS-induced colitis	(20)

*Hu, human; mo, mouse; mb, microbiota; UC, ulcerative colitis; CD, Crohn's disease; GWAS, genome-wide association study; IBD, inflammatory bowel disease; AOM, azoxymethane; IEC, intestinal epithelial cells; DSS, dextran sulfate sodium; TNBS, 2,4,6-trinitrobenzene sulphonic acid; mRNA, messenger RN; KO, knock-out*.

**Table 2:** Non-steroidal nuclear receptors.

**Nuclear receptor**	**Spec**	**Model**	**Study outcome**	**References**
CAR NR1I3 (protective)	hu	Biopsies	*CAR* expression is reduced in CD and UC patients	(21)
	mo	Chemical	Colonic CAR expression is reduced in DSS-treated mice; CAR*^−/−^* mice exhibit reduced healing following DSS exposure	(21)
FXR NR1H4/5 (protective)	hu	Biopsies	Reduced ileal *FXR* expression in CD patients	(22)
			Genetic variation of *FXR* is associated with IBD	(11)
	mo	Spontaneous	FXR protects the small intestine against bacterial overgrowth and the disruption of the epithelial barrier	(13)
		Chemical	*FXR^−/−^* mice are more susceptible to DSS and TNBS, enhanced levels of pro-inflammatory cytokines	(23)
			FXR protects against colitis symptoms (DSS and TNBS)	(24)
LRH-1 NR5A2 (protective)	hu	Biopsies	Reduced mRNA and protein expression of LRH-1 in CD and UC patients	(25)
	mo	Chemical	*LRH-1^+/−^* mice are more susceptible to DSS and TNBS colitis and show enhanced inflammatory responses	(25)
		AOM/APCmin	*LRH-1^+/−^* mice show reduced intestinal tumorigenesis	(26)
LXR NR1H3/2 (protective)	hu	GWAS	*LXR* polymorphisms contribute to enhanced risk of developing IBD	(27)
		Biopsies	Colonic *LXRα* and *LXRβ* expression is significantly reduced in IBD patients	(28)
		Cell culture	Loss of *LXR* expression and function is believed to reduce fatty acid synthase expression in UC patients	(29)
	mo	Chemical	*LXR^−/−^* mice are more susceptible to colitis (DSS and TNBS)	(28)
NUR77 NR4A1 (protective)	hu	GWAS	*NR4A1* gene locus is associated with an increased risk for UC and CD; reduced *NR4A1* expression in colons from patients	(30)
	mo	Chemical	NR4A1 expression is reduced in DSS colitis, *Nur77^−/−^* mice are more susceptible to DSS-induced colitis	(30, 31)
NUR1N R4A2 (protective)	mo	Chemical	Loss of NR4A2 in CD4 T cells only leads to an increased susceptibility to DSS-induced colitis	(32)
PPARα NR1C1 (protective)	mo	Chemical	*PPARα^−/−^* mice are more susceptible to TNBS colitis	(33, 34)
			PPARα controls aspects of colonic inflammation (DSS)	(35)
PPARδ NR1C2 (protective)	mo	Chemical	*PPARβ/δ^−/−^* mice are more susceptible to DSS-induced colitis	(36)
PPARγ NR1C3 (protective)	hu	Biopsies	*PPARγ* expression is reduced in UC patients	(37–40)
			*PPARγ* expression is decreased in intestinal samples from IBD patients	(41)
			SNPs in PPARγ are associated with CD	(42)
		GWAS	*PPARγ* polymorphism is associated with susceptibility to IBD	(27, 43)
	mo	Chemical	PPARγ protein levels are decreased during DSS colitis	(44)
			Intestinal epithelial cell-specific *PPARγ^−/−^* mice are more susceptible to induction of DSS colitis	(45)
PPARγ NR1C3 (protective)	mo	Chemical	IEC-specific deletion of PPARγ enhances colonic inflammation (DSS)	(46)
			Induction of DSS colitis in CD4^cre^ *PPARγ^*flfl*^* mice enhances disease severity and histopathology	(46)
			Macrophage-specific PPARγ deletion in mice significantly exacerbated DSS colitis	(47)
			*PPARγ^+/−^* mice are more susceptible to induction of TNBS colitis	(48)
			*PPARγ^+/−^* mice are more susceptible to induction of TNBS colitis	(49)
		Transfer	Treg-intrinsic PPARγ activation prevents colitis progression	(50)
		Ischemia	*PPARγ^−/−^* mice are more susceptible to tissue injury	(51)
		Spontaneous	*Pparγ* as a susceptibility gene in SAMP1/YitFc mouse Crohn's disease	(42)
			PPARγ induces colon epithelial expression of β-defensins and therefore functions as an antimicrobial factor	(52)
PXR NR1I2 (protective)	hu	Biopsies	*PXR* mRNA expression is significantly reduced in colons of UC patients (unaffected in CD)	(53)
			Decreased *PXR* expression in intestinal samples from IBD patients	(41)
			PXR is associated with IBD	(54–57)
		GWAS	Several PXR haplotypes contribute to CD susceptibility	(27, 58)
	mo	Chemical	PXR activation ameliorates DSS-induced colonic injury	(47, 59)
			Gut injury was more severe in *PXR^−/−^* mice challenged by experimental necrotizing enterocolitis	(60)
Rev-Erb α/β; NR1D1/2 (protective)	hu	Biopsies	NR1D2 expression is downregulated in UC patients	(61, 62)
RORα; NR1F1 (promotion)	hu	Biopsies	*RORα* expression is upregulated in colonic mucosa of CD patients	(62)
RORγt; NR1F3 (promotion)	mo	Transfer	Adoptive transfer of *RORγt*-deficient T cells into *Rag1^−/−^* mice failed to induce colitis	(63)
RXR; NR2B1,2,3 (protective)	mo	Chemical	*RXRα^+/−^* mice are more sensitive to TNBS and DSS-induced colitis	(49, 64)
VDR NR1I1 (protective)	hu	Biopsies	IBD susceptibility and VDR polymorphism are genetically associated	(52, 65–72)
			Colonic epithelial *VDR* expression was reduced in CD or UC patients	(73, 74)
			Vitamin D deficiency associates with an increased risk of IBD in epidemiological studies	(75–81)
	mo	Transfer	*VDR^−/−^* T cells induced enhanced colitis symptoms in *Rag1^−/−^* mice	(74, 82)
		Spontaneous	VDR/IL-10 d.k.o. mice developed accelerated IBD resulting in 100% mortality by 8 wks. of age	(82–84)
		Infection	*Salmonella* infection induced colonic epithelial VDR expression, and VDR attenuates responses to infection	(85, 86)
		Chemical	VitD deficiency predisposes mice to DSS colitis	(87)
			Intestine-specific *VDR^−/−^* mice developed enhanced DSS colitis (mucosal damage, increased pro-inflammatory cytokines	(86, 88)
			hVDR-expressing mice are highly resistant to DSS and TNBS-induced colitis	(74)
			*VDR^−/−^* mice are extremely sensitive to DSS colitis	(82, 89)

**Table 3:** Orphan nuclear receptors.

**Nuclear receptor**	**Spec**	**Model**	**Study outcome**	**References**
HNF4α NR2A1 (protective)	hu	Biopsies	*HNF4A* expression is decreased in intestinal samples from IBD patients	(41, 90, 91)
		GWAS	*HNF4A* locus is associated with an increased risk for UC	(92–95)
	mo	Chemical	IEC-specific *Hnf4α^−/−^* mice are more susceptible to DSS-induced colitis	(41)
		Spontaneous	Development of spontaneous colitis in aged mice	(90)
	mb	Meta-analysis	Interactions between HNF4α and microbiota gene expression patterns are associated with human IBD	(96)
NR2F6 EAR2 (protective)	hu	Biopsies	High *NR2F6* expression in healthy IECs, downregulated *NR2F6* expression in intestinal mucosa of IBD patients	(61, 97–100)
	mo	Chemical	*Nr2f6^−/−^* mice are more susceptibility to DSS induced colitis due to loss of barrier integrity and reduced Muc2 gene regulation	(101)
		Spontaneous	Spontaneous colitis phenotype in aged mice	(101)

*Hu, human; mo, mouse; mb, microbiota; UC, ulcerative colitis; CD, Crohn's disease; GWAS, genome-wide association study; IBD, inflammatory bowel disease; AOM, azoxymethane; IEC, intestinal epithelial cells; DSS, dextran sulfate sodium; TNBS, 2,4,6-trinitrobenzene sulphonic acid; mRNA, messenger RNA; d.k.o., double knock-out*.

**Table 4:** Human IBD therapy—clinical trials.

**NR**	**Compound**	**Mechanism**	**Cell type**	**References**
GR	Glucocorticoids	Anti-infl	ImC	(1–4)
	Prednisolone	Anti-infl	ImC	UC:(5–7)
				CD: (8, 9)
	Budesonide	Anti-infl	ImC	UC: (10–14)
	Prednisone	Anti-infl	ImC	CD: (15–17)
				UC: (18)
	Prednisolone	Anti-infl	ImC	(6, 19–21)
	Methylprednisolone	Anti-infl	ImC	CD: (11, 22)
	Beclomethasone	Anti-infl	ImC	CD: (13)
				UC: (23)
	Cortisone	Anti-infl	ImC	UC: (24)
	Fluticasone	Anti-infl	ImC	UC: (25–27)
PPARγ	5-ASA (Sulfasalazine, Mesalazine, Mesalamine)	Anti-infl	ImC	CD & UC: (28)
	Rosiglitazone	Anti-infl	ImC	UC: (29–31)
		Perm	IEC	UC: (31)
	5-ASA + Rosiglitazone	Anti-infl	ImC	UC: (32)
PXR	Rifaximin	Anti-mic	mb	(33–37)
RORyt	Secukinumab	Anti-inflam	ImC	(38)
VDR	Vitamin D	Anti-infl	ImC	(39)
		Perm	IEC	(40–44)
		Anti-infl	ImC	(45–51)
		Pro-bact	mb	(52, 53)

*Anti-infl, anti-inflammatory; pro-infl, pro-inflammatory; anti-mic, anti-microbial; pro-bact, pro-bacterial; perm, epithelial permeability; mb, microbiota; ImC, immune cells; IEC, intra epithelial cells; UC, ulcerative colitis; CD, Crohn's disease*.

In humans, the NR family consists of 48 members and is, therefore, the most significant group of transcriptional regulators. It includes the receptors for steroid and thyroid hormones together with receptors for lipophilic vitamins and cholesterol metabolites (6, 7). The physiological ligands for approximately half of NRs are known, whereas the rest are classified as orphan receptors (8, 15–17). Members of the NR family are highly conserved; the modular domain structure consists of an activation domain (AF), the central DNA-binding-domain (DBD), the hinge region, the ligand-binding domain (LBD), and the activation function 2 (AF2) (15). NRs differ in their modes of action. In the classical steroid receptor signaling, for instance, the ligand (steroid) enters the cell to activate the receptor located in the cytoplasm. Due to the resulting conformational change, the receptor translocates to the nucleus and binds to its cognate nuclear receptor response element on the DNA within the target gene promoters thereby altering the transcription levels (18). However, other nuclear receptors, such as thyroid hormone receptors or the peroxisome proliferator-activated receptors (PPARs), are localized in the nucleus regardless of whether or not they are bound to a ligand and constitutively interact with DNA response elements (1). Of note, additional non-genomic functions of nuclear receptors in the cytosol have been firmly established such as the activation of cAMP, Ca^2+^ or the MAPK signaling cascade (17–19). The specificity of transcriptional activation by a given NR is achieved by the tissue-selective expression of co-repressors or co-activators as well as post-transcriptional modifications of both.

Expression analysis of biopsies from IBD patients as well as animal studies with NR ligands suggests a significant correlation between NR biology and IBD pathology (Tables 1–4) (Figure 1). Interestingly, the presence of NRs or their ligand agonists seems to be mainly protective during IBD (Tables 1–3). The NR superfamily is one of the primary classes of therapeutic drug targets for human disease (1, 2). How NRs regulate gut homeostasis in the complex interplay between intestinal epithelial cells, the immune system, and the microbiota is an active area of research.

Ligands targeting NRs in IBD, either being tested in clinical trials or already in use to treat IBD patients, are dexamethasone and methylprednisolone (targeting GR), rosiglitazone, pioglitazone, bezafibrate, and curcumin (targeting PPARγ), and 1,25-di-hydroxyvitamin, calcitriol, and cholecalciferol (targeting VDR) (3, 4) (Table 4).

Current strategies to treat IBD include anti-inflammatory drugs, immunosuppressives, biological agents, antibiotics, and changes in dietary habits in combination with pain medication (20). These treatment options help relieve symptoms and reduce the risks of recurrence and complications, but in most cases, only a subgroup of patients responds to the available therapies. Surgery is the last therapeutic possibility when there is loss of response and adverse side effects. In the context of an increasing number of IBD patients, new approaches to treatment are needed, and molecular targets such as NRs represent a promising avenue to pursue in a search for more effective drugs. This review focuses on the complex relationship between nutrition, inflammation and nuclear receptor biology within the GI and the emerging number of NR ligands used in IBD therapy.

### Nuclear Receptors Regulate Intestinal Homeostasis

Nutrient uptake and elimination of toxic dietary components or xenobiotics within gut epithelium are dependent on the dietary lipid-activated NRs such as CAR (NR1I3), FXR, PXR, and VDR. Also, glucose, fatty acid, triglycerides, and lipoprotein metabolism in intestinal epithelial cells (IECs) are regulated by the PPAR family (α, β, δ), whereas cholesterol transport and absorption and bile acid metabolism are dependent on LXR and LRH (NR5A2) (21).

**Figure 2:**
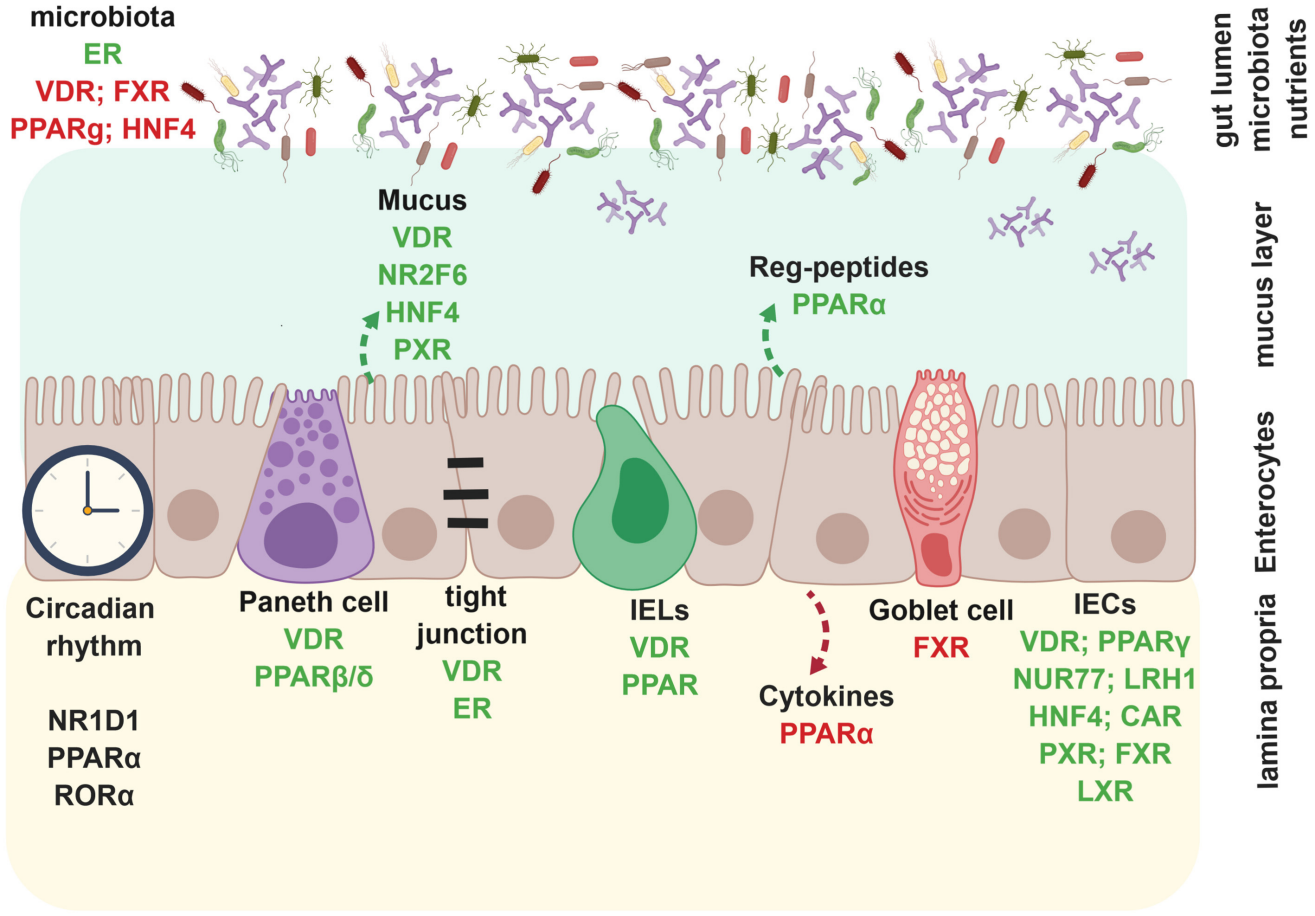
Nuclear receptors regulate intestinal epithelial barrier. During homeostasis, nuclear receptors such as VDR, FXR, PPARγ, HNF4α inhibit bacterial outgrowth whereas ER enhances microbiota richness. VDR, NR2F6, HNF4α, and PXR promote mucus secretion and epithelial barrier integrity, whereas VDR and ER directly enhance tight junctions. PPARα specifically promotes the production of anti-microbial Reg-peptides, beneficial for an intact barrier. Circadian rhythm in enterocytes is dependent on NR1D1, PPARα, and RORα. VDR and PPARβ/δ positively regulate paneth cell development. VDR and PPARα also promote CD8αα^+^ IELs. PPARα enhances expression of pro-inflammatory in enterocytes. FXR inhibits goblet cell development. VDR, PPARγ, NUR77, LRH-1, HNF4α, PXR, FXR, and LXR promote enterocyte development, whereas CAR is involved in wound healing of IECs. Created with BioRender.

Furthermore, NRs such as ERβ, RARα, HNF4α, and NR2F6 regulate essential aspects of intestinal barrier functions such as mucus secretion, goblet and paneth cell numbers, autophagy and expression of tight junction proteins (Figures 1, 2) (11–14, 22).

Microbiota, and their metabolites such as butyrate, propionate, or indole, influence NR biology directly, functioning as ligands to target FXR, PPARγ, or PXR (Figure 1) (5). Depletion of butyrate-producing microbes by antibiotic treatment reduces epithelial signaling through PPARγ showing that microbiota-activated PPARγ signaling prevents the dysbiotic expansion of potential pathogens (23) (Figures 1, 2). However, FXR activation itself alters the intestinal microbiota and could provide opportunities for microbiome biomarker discovery or new approaches to engineering the human microbiome (24–26) (Figure 2). For detailed aspects of nuclear receptor and microbiota biology, we refer to a recent review by Duszka and Wahli (27).

Within the intestinal epithelium, NRs such as VDR, HNF4α, LXR, PPARγ, LRH1, and NR2F6 play protective roles in intestinal epithelial integrity (Figures 1, 2); decreased mRNAs have also been validated in intestinal samples from IBD patients (10, 12) (Table 1). In mice, deletion of the VDR increases mucosal injury that leads to high mortality in DSS-induced experimental colitis (10). In parallel, the activation of the farnesoid X receptor (FXR) prevents chemically-induced intestinal inflammation, improves colitis symptoms, inhibits epithelial permeability, and reduces goblet cell loss (13) (Figure 2). Intestinal steroidogenesis controls PPARγ expression in the colon, and this axis is impaired in ulcerative colitis (11).

The microbiota-NR axis influences not only metabolism of the intestinal epithelium, but also the components of the circadian clock; in particular, RORα (NR1F1) and RevErbα (NR1D1) influence corticosterone synthesis in IEC whereas PPAR and LXR families can alter the hepatic circadian clock (28, 29) (Figure 2).

NRs contribute especially to gut homeostasis by shaping intestinal immune cells; on one side, they are constantly challenged in the face of stimulatory signals from nutrients and gut microbiota, and on the other, they shape the composition of the microbiota themselves (Figures 1, 2) (5, 13, 19, 28, 29). Already the development of gut-associated lymphoid tissue is dependent on the expression of NRs like RORγt (NR1F3), which is required for the generation of lymphoid tissue inducer (LTi) cells and subsequent formation of Peyer's patches. As the amount of RORγ protein is reduced in the absence of the vitamin A metabolite retinoic acid (RA), this suggests that RAR directly controls the fetal development of intestinal secondary lymphoid organs (SLOs) as well as the fitness of the immune system in adulthood [recently reviewed (19)].

**Figure 3:**
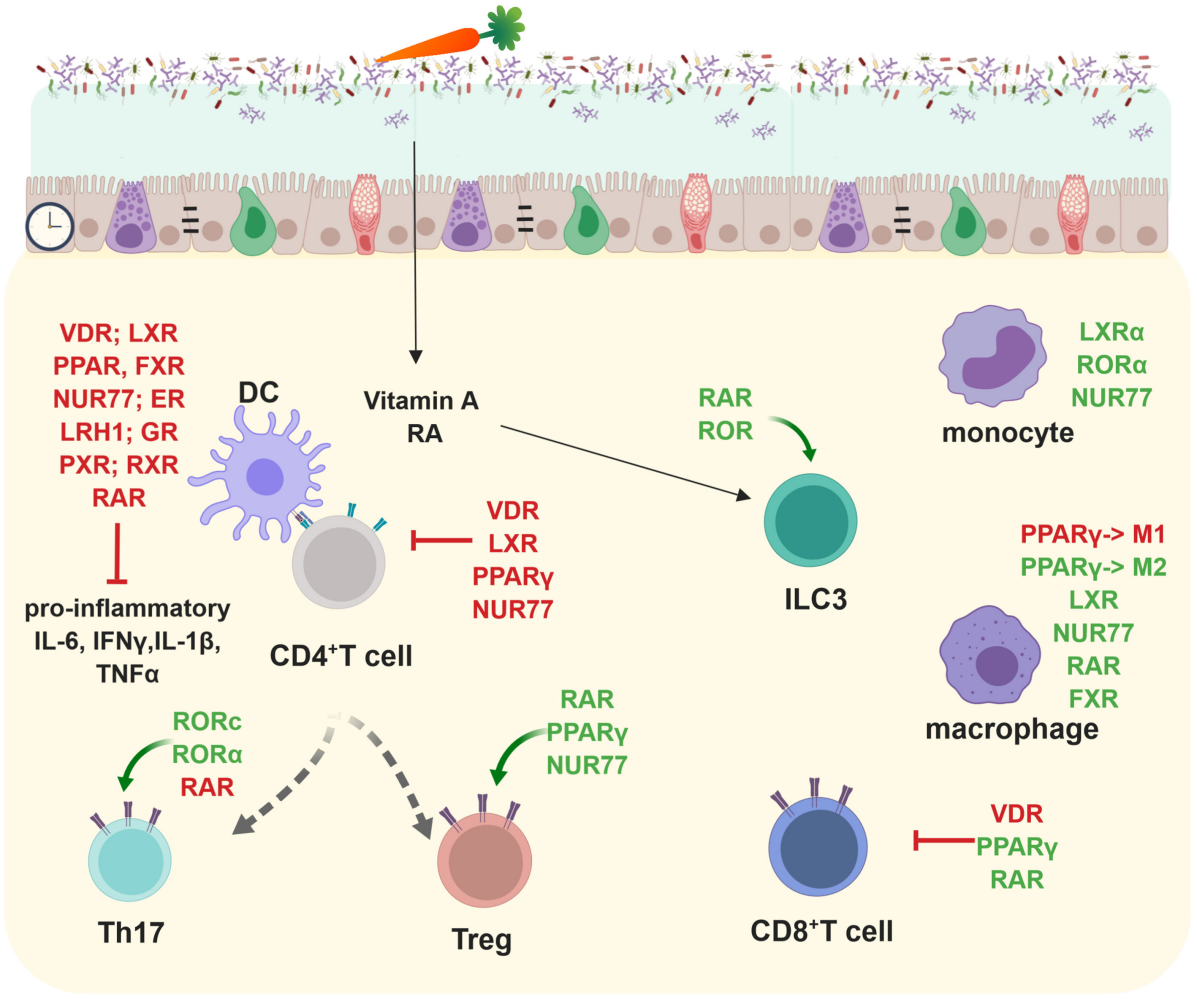
Nuclear receptors shape adaptive and innate immune responses in the lamina propria and subsequently gut homeostasis. Most NRs (VDR, LXR, PPAR, FXR, NUR77, ER, LRH-1, GR, PXR, RXR, and RAR) target pro-inflammatory cytokine production (IL-6, IFNγ, IL-1β, TNFα). RORc and RORα are important in promoting Th17 development whereas RAR, PPARγ, and NUR77 positively influence regulatory T cells. VDR, LXR, PPARγ, and NUR77 inhibit CD4^+^ T cell responses. RAR and ROR promote ILC3 differentiation. LXRα, RORα, and NUR77 positively influence monocytes. PPARγ inhibits M1 macrophages but promotes M2 macrophages which are important anti-inflammatory effector cytokine producers. LXR, NUR77, RAR, and FXR foster in parallel the anti-inflammatory responses of macrophages. VDR enhances inflammatory whereas PPARγ, and RAR suppresses pro-inflammatory CD8^+^ T cell responses. Created with BioRender.

Macrophages expressing LXR, NR4A1 (NUR77), PPARγ, or RARα are essential for gut immune homeostasis (30–34) (Figure 3). Especially the reciprocal differentiation potential of naïve CD4^+^ T cells into either pro-inflammatory Th17 or tolerance-inducing regulatory T cells is dependent on several NRs such as RORγ, RORα, LXR, NR4A2 (NURR1), PPARγ, RAR, or VDR (35) (Figure 3). Whereas, RAR-related orphan receptors (RORγ and RORα) are key transcriptional activators, RAR, RXR, NUR77, PPARγ, LXR, GR, VDR, and ER contribute to anti-inflammatory effects (31, 32, 36–40) (Figure 3).

The NR network in CD4 Th17 cells is highly complex. PPARγ suppresses Th17 differentiation by directly interfering with the silencing mediator of retinoid acid and thyroid hormone receptor (SMRT) clearance from the *Rorc* promoter. PPARγ activation suppresses not only the expression of *Rorc* but also subsequently that of *Il17a, Il17f*, *Tnfa, Il22, Il21, Il23r, CCR6*, and *CCL20* (18). LXR reduces the expression of *Ror*γ*t, Il17a, Il17f, Il22, Il23r*, and *Ahr* but does not affect that of *Il21* and *Rora*. RAR suppresses Th17 differentiation through retinoic acid-mediated inhibition of *Il23r, Il6r*, and *Irf4* expression and Smad2/3 phosphorylation via the TGFβ receptor pathway (43). NR2F6 directly binds to RORE sites within the *Il17a* locus and subsequently interferes with the transactivation of the *Il17a* promoter by RORc (41). It is still unclear how specificity is achieved despite highly homologous hormone response (HRE) DNA-binding core consensus sequences.

NRs, such as VDR or PPAR also regulate intestinal CD8 T cell responses (42–44). In parallel to the CD4 Th17 compartment, NR of the retinoic acid and retinoic acid-related family are key regulators of innate lymphoid cells (ILCs).

Innate lymphoid cells are tissue-resident immune cells that play essential roles in maintaining and protecting the gastrointestinal barrier against invading pathogens. In particular, ILCs themselves mediate immune responses but are controlled by dietary components and microbial metabolites. ILCs also directly regulate host metabolism and glucose tolerance (45). NR of the retinoic acid and retinoic acid-related family are key regulators of group ILC2 and 3 (46–48) (Figure 3). In a healthy state, ILC3 are responsible for mucosal homeostasis through the secretion of moderate amounts of IL-22, IL-17, and GM-CSF (49). Especially, regulation of gastrointestinal homeostasis and dysregulation by ILC3s result in overexpression of the IL-17, IFNγ, and IL-22 pro-inflammatory cytokines which can be seen in mice and IBD patients (50–57).

Thus, whereas on one side, microbiota and their metabolites shape the homeostasis of the gut immune system, innate and adaptive lymphocytes sequentially shape gut microbiota and lipid metabolism on the other. Thus, NRs occupy a center stage of gut immune homeostasis (27).

### Diseases of the Gastrointestinal Tract

The two major forms of chronic inflammatory disorders within the gastrointestinal tract are Crohn's disease (CD) and ulcerative colitis (UC), characterized by clinical symptoms like severe diarrhea, pain, fatigue and weight loss (58). UC primarily affects the colon and rectum, whereas CD targets the small and large intestine, the mouth, esophagus, stomach, and the anus (59, 60). The etiology of the disease is multifactorial including genetic predisposition, the composition of gut microbiota, and environmental factors such as nutrition and antibiotic usage which subsequently can also alter immune responses (61). Intestinal barrier integrity is one of the most critical factors for a healthy GI tract, as an invasion of solutes, microorganisms and luminal antigens cause immune cell infiltration and inflammatory responses (62). Treatment options such as the corticosteroid prednisone or the anti-tumor necrosis factor-α antibody Infliximab suppress the immune system and relieve symptoms of patients.

Several members of the NR family have a protective role during disease progression, and their loss in different IBD animal models (DSS, TNBS, T cell transfer, or infection) leads to exacerbated colitis symptoms (Tables 1–3) (Figure 1). In human genome-wide association studies (GWAS), several nuclear receptor polymorphisms have been associated with IBD, and NR expression is mostly down-regulated in biopsies from UC and CD patient in comparison to healthy subjects (12, 63–73) (Tables 1–3). As IBD onset typically occurs in the second and third decade of life with a high number of patients progressing to relapse and chronic disease, an urgent need to develop new therapies with either low adverse side effects during long-term management or even curative potential is needed in the future.

### Nuclear Receptors as Therapeutic Targets in the Clinic

The characterization of NRs that either promote or suppress intestinal inflammation has led to efficacious therapeutics for IBD. One classic anti-inflammatory drug, namely 5-ASAs, augments PPARγ expression and promotes its translocation from the cytoplasm to the nucleus resulting in activation of peroxisome-proliferator hormone response element-driven genes to suppress colitis activity (74, 75). Regarding combinatorial therapy, the treatment with 5-ASAs and rosiglitazone (PPARγ agonist) had a better therapeutic effect in UC than 5-ASA alone (76–78) (Table 4).

Regulating the glucocorticoid receptor (GR), glucocorticoids play an important role in inducing remission in IBD (79). Unfortunately, response rates are low and vary between 20 and 30% of patients showing resistance with the therapy also inducing common side effects (80) (Table 4).

There is strong evidence in support of vitamin D that targets the VDR having protective effects in IBD-related inflammatory responses (81). Treatment with vitamin D3 in mild to moderate CD patients significantly improved disease activity and quality of life after 24 weeks of treatment (Table 4) (82). More studies showed a positive effect of vitamin D supplementation in UC and CD patients (82–86). However, since the clinical efficacy and mechanism of action of vitamin D therapy are unclear, additional studies are necessary to fully explore its possible immunomodulatory and anti-inflammatory effects, also in relation to decreasing epithelial permeability and maintaining barrier integrity (87) (Tables 2B, 4).

Rifaximin, an intestine-specific human PXR agonist, appears to have more antimicrobial efficacy in the therapy of CD than traditional medications like metronidazole or ciprofloxacin; it decreases intestinal permeability and targets (NFκB), regulating anti-inflammatory effects in IBD patients (88–90) (Table 4).

Besides clinical data on nuclear receptor therapy targets, so far, several NRs have only been tested pre-clinically in animal models.

### Steroid Hormone Receptors

#### Estrogen Receptor (ER; NR3A)

The estrogen receptors, members of the steroid hormone receptor family, play an essential role in the maintenance of colonic homeostasis. Understanding the biological effects of ERα and ERβ within the gut and the immune system are important for unraveling the gender-dependent differences in intestinal inflammatory diseases. Interestingly, estrogen levels impact the composition of gut microbiota itself (91); however, the composition of the microbiota influences the bioavailability of estrogen (92) (Figure 2). Males are at greater risk than females for developing ulcerative colitis (UC) and experiencing worse clinical progression, whereas females are more likely to develop CD (93–96). Especially ERβ is expressed abundantly in the colonic epithelium, where it regulates maintenance of colonic architecture, tight-junction formation, and barrier function (97, 98). *ER*α and *ER*β gene expression levels are comparable between male and female UC colon samples, suggesting that sex-based differences in ER-mediated effects are most likely not caused by differences in gene expression (Table 1) (99).

Nevertheless, *ER*β expression has been found markedly decreased in colonic mucosa of CD/UC patients with active disease; specifically, *ER*β expression in female mice protected against DSS colitis, whereas it failed to protect male mice (100). Recently, Wendy A. Goodman et al. (93) reported that fundamental differences in ERα/ERβ signaling ratios impact colitis in males and females. Analysis of gene expression from inflamed colonic tissues identified alteration of typical estrogen-responsive genes such as *Socs3, Ctsd*, and *Fos* as being up-regulated in colon tissues of DSS-treated *ER*α-knockout male mice compared with *ER*α-knockout females. In line with these data, similar gene expression profiles of *SOCS3, CTSD*, and *FOS* were found in colonic biopsy specimens from male and female patients suffering from UC (93, 99) (Table 1).

Experimental colitis studies in mice and rats have shown that ER has pre-clinical therapeutic implications (93, 101–103). Supraphysiological doses of 17β-estradiol have anti-inflammatory (in the DNB mouse colitis model) as well as pro-inflammatory (in the DSS mouse colitis model) effects demonstrating complex immunomodulation in female mice during intestinal inflammation (102). Additionally, studies in male rats and in the HLA-B27 transgenic rat IBD model demonstrated reduced colonic damage score with estradiol treatment during acute colonic injury (101, 103).

#### Glucocorticoid Receptor (GR; NR3C1)

Glucocorticoids targeting the GR contribute to diverse biological processes including glucose metabolism, stress, or immune responses. Endogenous GCs are predominantly produced by the adrenal glands, but within the IEC the NR LRH-1 regulates extra-adrenal glucocorticoid synthesis in the intestine (104).

The GR is expressed in almost every cell in the body and is a multi-tasking transcription factor, changing its role and function from anti-inflammatory effects via direct gene suppression or activation to potential pro-inflammatory actions as well [reviewed in (105)]. Nevertheless, rapid non-genomic mechanisms of GC signaling have also been reported [reviewed in (105)].

A mechanism for glucocorticoid-mediated inhibition of immune responses is the interference with activities and modulation of key pro-inflammatory transcription factors, including NF-κB, activator protein 1 (AP-1), members of the signal transducer and activator of transcription (STAT), CCAT/enhancer-binding protein (C/EBP), and nuclear factor of activated T cells (NFAT) families (106, 107). Through GR-mediated transrepression, expression of pro-inflammatory cytokines and chemokines like IL-1α, IL-1β, and IL-8 are down-regulated (Figure 3). Additionally, the GR can directly activate suppressive inflammatory mediators like TGF-β and IL-10, inhibit T and B lymphocyte proliferation, and promote a tolerant macrophage profile (M2), altogether increasing its anti-inflammatory function (105, 108) (Figure 3). The signaling pathways of GR and PPARα, another nuclear receptor, can cooperate and increase the inhibition of cytokine gene expression to alleviate inflammation (109).

Within the immune system, glucocorticoids are circadian mediators (110) and regulate diurnal oscillations in T cell distribution by inducing IL-7R and CXCR4 (111) and regulate T cell responses in gastrointestinal Peyer's patches. Dexamethasone suppresses IL-23–mediated IL-22 production in human and mouse ILC3s (112).

GCs have a long history in IBD therapy and are well-known immune suppressants. Nevertheless, the expression of the GR itself does not appear to predict steroid treatment responses in IBD patients although conflicting data exist (Table 1). GCs are especially able to protect mice and men against TNF-induced inflammatory symptoms, and GR dimers control intestinal STAT1 and TNF-induced inflammation in mice (113, 114).

The role of the GR in IBD has been reviewed recently (115). Human trials demonstrated that standard systemic corticosteroids (cortisone, prednisone, methylprednisolone, fluticasone) are effective in inducing remission in UC by suppressing immune responses, and might be of benefit in CD (3) (Table 4). Therefore, glucocorticoids are still the mainstay for induction of clinical remission in cases of acute relapse of both CD and UC, and second-generation corticosteroids such as budesonide or beclomethasone have been developed. Whereas, budesonide induces remission in active ileal CD, it shows less efficacy in and does not prevent CD relapse (3, 116) (Table 4). Many preclinical studies were performed using different IBD animal models to investigate the complex cellular and molecular basis of glucocorticoid action at the interface between the endocrine, the immune, and the intestinal system. As a future perspective, screening assays for GR agonists are ongoing in order to develop new effective medications against acute inflammation (117).

The role of other steroid hormone receptors such as the androgen receptor (AR) and the progesterone receptor (PR) during colitis progression have been investigated only poorly. Pre-diagnostic circulating testosterone is associated with a lower risk of CD but not UC in women (95). Progesterone therapy decreases oxidative damage, characterized by decreased MDA, MPO, TNFα and caspase-3 activity, in the colonic mucosa (118).

#### Non-steroidal Nuclear Receptors

Despite a wide range of pre-clinical IBD trials with compounds specifically targeting NR family members, the following NRs have not yet reached UC or CD patients in clinical trials.

#### Constitutive Androstane Receptor (CAR; NR1I3)

The xenobiotic NR CAR can be regulated by xenobiotics and endobiotics but also by steroid hormones (119). One of its diverse metabolic functions (119) includes the clearance of xeno- and endobiotics such as toxic bilirubin (Table 2A) (120). Its expression in the intestine and the liver is dependent on the presence of microbiota (119, 121, 122).

CAR is expressed in the healthy intestinal epithelium, but the expression is reduced within intestinal mucosal biopsies from patients with UC and CD, or tissue from DSS mice (67, 123) (Figures 1, 2). In the pre-clinical DSS mouse model, especially wound healing of intestinal epithelial cells is reduced in *Car*-deficient mice whereas activation of CAR using a selective CAR agonist 3,3',5,5'-tetrachloro-1,4-bis(pyridyloxy)benzene (TCPOBOP) enhances mucosal healing (67) in mice (Figure 2). In a rat DSS colitis model, CAR agonists reduced the mRNA expression of several pro-inflammatory cytokines in a CAR-dependent manner; CAR inhibited apoptosis by inducing Gadd45b within an *in vitro* cell analysis (124). Therefore, CAR activation may also prove effective in patients with IBD.

#### Farnesoid X Receptor (FXR)

FXR functions as an enterohepatic regulator of bile acid homeostasis and regulates especially lipid (125) and glucose metabolism (126), as well as inflammation (13). *Fxr*-deficient mice are more susceptible to IBD models such as TNBS or DSS due to enhanced expression of pro-inflammatory cytokines in innate immune cells (127) (Table 2A) (Figures 1, 3). Also monocytes and dendritic cells (DCs) are modulated by FXR and there is a decrease in epithelial expression of pro-inflammatory molecules both *in vivo* and in stimulated epithelial cultures after induction of FXR signaling, suggesting that the immunomodulation observed might be partly mediated through epithelial effects (13, 128) (Table 2A; Figure 2). Along with the FXR ligand, INT-747 represses the expression of various pro-inflammatory cytokines, chemokines and their receptors (13, 127). Colon inflammation in CD patients and rodent models of colitis is associated with reduced expression of *FXR* mRNA (Table 2A) (127). FXR also regulates gut barrier function due to its antibacterial growth effect (129) and its control of proliferating Lgr5^+^ intestinal stem cells (130) (Figure 2). Bile acids are well known natural ligands of FXR and regulate the protective activity of FXR in shielding the intestine from bacteria-induced damage and thereby maintaining a competent gut barrier and preventing the development of IBD (Table 2A) (13, 131).

Several pharmacological modulators of FXR activity have been tested in human clinical trials, but its role in IBD has so far only been investigated in pre-clinical mouse models (13). Fexaramine is an intestinal-specific FXR modulator which is potentially safer than systemic FXR agonists as it preferentially activates FXR target genes in the intestine (132), but its functional role has not yet been investigated in IBD models.

#### Liver Receptor Homolog-1 (LRH-1; NR5A2)

LRH-1 is mostly known for its regulatory role in cholesterol and bile acid homeostasis but has recently emerged as a key regulator of intestinal function. Unlike most of the other NRs, LRH-1 acts constitutively to drive the transcription of its target genes (133). Nevertheless, this atypical NR contains a well-ordered hormone-binding pocket, which binds signaling phospholipids including phosphoinositides (134, 135).

LRH-1 is expressed in intestinal crypts, where intestinal stem cells (ISCs) reside, and where it contributes to epithelial renewal by potentiating WNT/β-catenin signaling (136–138). GWAS meta-analyses of IBD patients found a significant association between LRH-1 and IBD (Table 2A). Subsequent analysis on IBD patients revealed a significant decrease in expression of *LRH-1* and its transcriptional targets such as *CYP11A1* and *CYP11B1* in the affected tissues (69, 139). Both *Lrh-1* haploinsufficiency and somatic deficiency of *Lrh-1* in the intestinal epithelium rendered mice more susceptible to experimentally induced DSS or TNBS colitis (Table 1) (69). One pathway how LRH-1 limits inflammation involves the regulation of extra-adrenal glucocorticoid production in the gut (69, 133). Apart from the immune-regulatory action on local immune cells, glucocorticoids may also induce intestinal tight junction proteins and improve epithelial barrier function (Table 1; Figures 1, 2) (140). It is plausible that after hapten-induced mucosal inflammation, the cell cycle regulatory function of LRH-1 comes into play to promote mucosal renewal and regeneration (69). A recent study also underpins the human relevance, using humanized mouse intestinal organoids, a humanized *in vivo* IBD model, and human intestinal organoids (Table 2A). Thereby, Bayrer et al., uncovered an essential role for LRH-1 in intestinal epithelial homeostasis and cell survival, which mitigates inflammatory injury (135). As preliminary therapeutic results, the use of DLPC (dilauroyl phosphatidylcholine) as an extrinsic agonist ligand for LRH-1 has been reported to result in decreased colitis symptoms (69).

#### Liver X Receptors (LXR; NR1H)

LXRs control lipid and glucose homeostasis and respond to physiological concentrations of sterols. Whereas, LXRα is mainly expressed in the liver, intestine, kidney, and immune cells, LXRβ is more ubiquitously expressed (141–143). Within the immune system, both LXRs are important anti-inflammatory transcription factors and physiological regulators of innate and adaptive immune responses, apoptosis, and phagocytosis (144).

Several LXR agonists are effective in pre-clinical models of diseases such as atherosclerosis or diabetes and are used as an anti-inflammatory agent (143, 145). *Lxr-*deficient mice are more susceptible to DSS colitis, show slower recovery and decreased survival (Table 2A). Expression of both *LXRA* and *LXRB* is significantly suppressed in the inflamed colon from both CD and UC patients compared with a non-inflamed colon (70), and LXR polymorphism has been linked to enhanced IBD risk (Table 2A) (146). While LXRα induces anti-inflammatory effects in innate immune cell populations (Figure 3), LXRβ has anti-inflammatory effects in colon epithelial cells (Figure 2). Addition of an LXR agonist GW3965 results in faster recovery and increased survival in pre-clinical mouse colitis models, making LXRs an exciting target to suppress inflammatory responses in IBD (70).

#### NR4A Family: (Nur77; NR4A1, Nurr1; NR4A2 and Nor1; NR4A3)

The orphan NR4A subfamily includes three members, which are expressed in a wide variety of tissues, especially innate and adaptive immune cells (147).

Although NR4A family members belong to the nuclear receptors superfamily, their activity is not considered to be regulated by physiological ligands, because their ligand-binding pockets are hidden by bulky amino acids, and their ligand-binding domains are constitutively active (148). Nevertheless, structurally diverse synthetic ligands for NR4A2 and NR4A3 have recently been identified (147). Genetic variants of the *NUR77* gene locus are associated with increased risk for both UC and CD, and *NUR77* expression is significantly reduced in colon tissues from patients with UC or CD and mice treated with DSS (Table 2A) (31, 149). *Nur77*-deficiency increases the susceptibility of mice to DSS and TNBS colitis and prevents intestinal recovery (31, 149) (Table 2A) (Figure 1). Mechanistically, NUR77 negatively regulate the TLR–IL-1R signaling axis (149). An independent study demonstrated that loss of *Nur77* in mice leads to enhanced colon inflammation with larger numbers of infiltrating neutrophils, T-cells, and macrophages during DSS colitis. *Nur77* overexpression dampens the pro-inflammatory state of both RAW macrophages and epithelial Caco-2 cells (Table 2A) (31).

The family member NR4A2 also regulates immune cell function and subsequently colitis, as deletion of NR4A2 in T cells attenuates induction of Tregs and causes aberrant induction of Th1 CD4^+^ T cells and subsequent exacerbation of colitis (Figure 3) (150). Treatment with cytosporone B (Csn-B), an agonist for Nur77, significantly attenuated excessive inflammatory response in mouse DSS colitis. Therefore, Nur77 has been suggested as a potential target for the prevention and treatment of IBD (149).

#### Peroxisome Proliferator-Activated Receptors (PPARγ, NR1C3)

PPARs are involved in the control of energy metabolism, inflammation and immune responses activated by natural ligands such as fatty acids, eicosanoids, and phospholipids (151, 152). PPARγ is highly expressed in both IECs and immune cells. Impaired epithelial expression has been documented in preclinical animal models of IBD and UC patients (Tables 2A,B) (Figures 1, 2).

Expression and activity of PPARγ are directly induced by microbial metabolites such as butyrate or propionate (153, 154).

Direct targeting of the activity of PPARγ to enhance anti-inflammatory effects via its agonistic ligand rosiglitazone is efficacious in the treatment of mild to moderately active UC (78, 155, 156) (Table 4). In combination with the anti-inflammatory drug 5-ASA (5-Aminosalicylate and its generics), rosiglitazone achieves better therapeutic effects without causing side effects in UC patients (76). Therefore, rosiglitazone is the most widely used therapeutic agent in conjunction with PPARγ activation that directly leads to trans-repressing of several pro-inflammatory target genes such as NF-κB and signal transducers and activators of transcription (STATs) (77, 157–161) (Figure 3). Also, microbiota-activated PPARγ signaling prevents dysbiotic expansion of potential pathogens by reducing the bioavailability of respiratory electron acceptors in the lumen of the colon (23) (Figure 2). Accordingly, several preclinical studies in IBD animal models have been and are being performed to investigate the molecular mode of action of new compounds that target PPARγ to enhance its anti-inflammatory effects within the immune and the epithelial compartment (Tables 2A,B) (162).

In addition to PPARγ, the two other family members, PPARα (NR1C1) and PPARδ (NR1C2) are used for therapeutic IBD intervention. Each PPAR isotype has a specific expression pattern within the gut, but all of them mediate the effects of the microbiota (163). Agonistic ligands targeting PPARα such as dexamethasone (164), fenofibrate (165), palmitoylethanolamide (166), or bezafibrate (167) have anti-inflammatory effects in pre-clinical animal studies. The first-generation PPARα agonists, the fibrates, have however been hampered by drug-drug interaction issues, statin drop-in, and ill-designed cardiovascular intervention trials reviewed in Bougarne et al. (168). Ambiguous results were obtained targeting PPARδ with GW0742 or dietary punicic acid showing either anti-, pro-inflammatory or no effects in experimental mouse IBD models (Tables 2A,B) (165, 169, 170).

#### Pregnane X Receptor (PXR; NR1I2)

PXR protects the body from harmful foreign toxicants and endogenous toxic substances as it induces genes involved in drug transport and metabolism (171). Pregnane X receptor is primarily expressed in the liver and the intestine; the distribution and function of human PXR in the gastrointestinal system contribute to its emerging role as a modulator of inflammation and the intestinal mucosal barrier (Table 2B) (171, 172) (Figures 1, 2). In contrast to most other NRs, PXR has a wide spectrum of ligands such as drugs, endogenous ligands or products of the gut microflora (173–175). Despite high homology between the LBD and DBD domain of human and mouse PXR, species-specific responses to ligand activation (such as rifampicin, or pregnenolone-16α-carbonitrile) are surprisingly different making results of pre-clinical mouse studies less extrapolatable for human trials (171).

PXR agonists reduce the mRNA expression of several pro-inflammatory cytokines in a PXR-dependent manner such as TNF-α and IL-1β in a rat DSS colitis model (124). PXR has been implicated in the pathogenesis of IBD, and its activator rifaximin (works only in humans, not in mice) has demonstrated efficacy in CD and UC (Table 2B). Antibiotic therapy with rifaximin, which was approved in 2004 for the treatment of traveler's diarrhea (176), was associated with the induction and maintenance of remission (90, 177). In CD patients, bile acid malabsorption is associated with deactivation of PXR (178). Despite the difference between human and mouse PXR species-specific responses, a lot of effort was made to test different ligands of PXR in mouse models of colitis utilizing its anti-microbial effects and its anti-inflammatory potential on immune cells as well as epithelial cells, helping the latter to maintain an intact epithelial barrier in the gut (Figure 3).

#### Retinoic Acid Receptor (RAR; NR1B)

RARs usually form heterodimers and function as ligand-dependent transcription factors but also play extra-nuclear and non-genomic roles. The vitamin A metabolite all-trans-retinoic acid (atRA) acts as a ligand for RAR and is involved in the regulation of both the intestinal barrier function as well as immune homeostasis (19, 179, 180).

In general, the NRs of the RAR family play important pleiotropic roles in the regulation of innate immune cells such as dendritic cells, macrophages, and ILCs, and are especially important in the regulation of T cell homing to the gut as well as IgA class switching in B cells (Figure 3) (19). The pleiotropic roles of retinoic acid and RARs as modulators of the immune system, for example, induction of Th1, Th2, and Th17 responses together with the release of pro-inflammatory cytokines like IL-12 and Il-23 by DCs, have been reviewed in much detail recently and will therefore not be discussed here (19, 30).

The vitamin A metabolite retinoic acid (RA) can also enhance ILC3 responses in mice through multiple mechanisms, including direct binding to the *Rorc* or *Il22 loci*, promoting maturation of LTi-like ILC3s, and regulating ILC3 proliferation (47, 51, 181). In addition to promoting maintenance of the intestinal epithelium, during fetal development vitamin A and the metabolite RA control the size of secondary lymphoid tissues via LTi cells in mice, which can influence the efficiency of protection from viral infections later in life (47, 51, 182).

In mouse or rat colitis models Vitamin A inhibits the development of DSS colitis and colon cancer (183, 184) (Table 2B). Several pre-clinical studies in mice show anti-inflammatory effects of RAR ligands like ATRA (185–187), Neomangiferin (188), 13cis-retinoic acid (189), or RA (44, 190). Importantly, atRA supplementation reduced the tumor burden in a mouse model of colorectal cancer via enhancing protective CD8^+^ T cell responses highlighting the relevance of NRs as a potential therapeutic option to treat colon cancer patients (191) (Figure 3).

#### RAR-Related Orphan Receptor Gamma (RORγ; NR1F3)

RORγ has a broad pattern of expression but is observed at very high levels within the thymus. There RORγ regulates thymocyte and lymphoid development but is also involved in the regulation of metabolism and the circadian rhythm (192). The recently de-orphanized RORγ is known to bind to sterols, with certain oxysterols having a very high affinity for this receptor. Synthetic inverse agonists of RORγ are effective in treating and preventing autoimmunity in mouse models and are beneficial in glucose and lipid metabolism (1). A cross-talk between RORγt^+^ ILCs and intestinal macrophages induces mucosal IL-22 production in Crohn's disease (193). In CD4^+^ Th17, the splice isoform RORγt controls the secretion of the cytokines IL-17a and IL-17f. Especially IL-17f has recently been identified as highly pathogenic in gut inflammation. Therefore, RORγt-expressing Th17 cells induce murine chronic intestinal inflammation (Table 2B) (194, 195). In parallel, delivery of IL-15 to CD4^+^ T cells in the colon downmodulates Foxp3 expression and enhances RORγt expression rapidly triggering IBD characterized by enhanced production of pro-inflammatory cytokines (such as interferon-γ, IL-6) and accumulation of Th1/Th17 cells (196) (Figure 3). Pharmacologic inhibition of RORγt via GSK805 provides therapeutic benefit in mouse models of intestinal inflammation and reduces the frequency of Th17 cells isolated from primary intestinal samples of individuals with inflammatory bowel disease (IBD) (197). In the course of IBD, RORα-dependent ILC3 functions are pivotal in mediating gut fibrosis, and they can offer an avenue for therapeutic intervention in Crohn's-like diseases (198).

#### Retinoid X Receptor (RXR; NR2B)

RXRs have been implicated in a diversity of cellular processes. These pleiotropic effects originate from the ability of RXRs to dimerize with diverse NRs, which exert transcriptional control on specific aspects of cell biology, and the ability to stimulate transcriptional activation by RXR partner receptors (199). RXRs form heterodimers, either spontaneously or in a ligand-dependent manner, with NRs well known to play crucial roles in the regulation of intestinal homeostases such as VDR, PPAR, FXR, LXR, or CAR (199) (Figure 2). Especially the RXR/PPARγ heterodimers, which are permissive to activation by both PPARγ and RXR ligands, have been investigated in colitis models (200). *Rxra*^+/−^ mice are highly sensitive to TNBS colitis and AOM/DSS colitis induction (Table 2B) (201). In the colon, the RXR ligand LG101305 is equally effective as PPARγ ligands in reducing intestinal inflammation during TNBS colitis. Also, rexinoids have a marked synergistic effect with PPARγ agonists on inflammation suggesting that co-administration of low doses of PPARγ and RXR agonists might be worth exploring in human IBD (200) (Table 4). Conventional full RXR agonists are known to show considerable adverse effects, but the partial RXR agonist, CBt-PMN, efficiently ameliorated the symptoms of colitis. This effect is attributed to the down-regulation of pro-inflammatory cytokines such as *Tnf* and *Il6* in colon-infiltrating monocytes probably by the activation of PPARδ/RXR and Nur77/RXR heterodimers by CBt-PMN (202).

#### Vitamin D Receptor (VDR, NR1I1)

In the intestine, VDR signaling regulates microbial homeostasis, barrier integrity as well as immune cell distribution and function (Table 2B) (203). The immune system, in particular, is influenced by vitamin D3 via enhancement of chemotactic and phagocytic responses of macrophages and production of antimicrobial proteins, such as cathelicidin; it inhibits the surface expression of the MHC-II-complex antigen and costimulatory molecules and downregulates the production of many pro-inflammatory cytokines, such as IL-1, IL-6, IL-8, and TNF-α (204). Association studies showing a higher incidence of CD follow a “North-South gradient” and support animal and clinical data demonstrating an important role for vitamin D as a risk factor and potential therapeutic target in CD (205) (Table 2B).

IL-22 production is dependent on vitamin D and in the absence of vitamin D, mice develop a more severe enteric infection that takes longer to resolve (Figure 2) (206).

In contrast Chen et al., report that VDR KO mice have more interleukin-22 (IL-22)-producing ILCs and more antibacterial peptides than WT mice. The increased ILCs in the VDR KO mice was a cell-autonomous effect of VDR deficiency on ILC frequencies (207).

A double-blind, randomized placebo-controlled study of the effect of vitamin D supplementation over 3 months showed significantly increased 25(OH)D levels in patients in remission accompanied by maintenance of the intestinal permeability (86) (Table 4). Several therapeutic studies in experimental IBD animal models and patients have subsequently shown vitamin D to have therapeutic efficacy on epithelial permeability as well as anti-inflammatory properties, and the effects of different analogs have been summarized in a recent review (203) (Figure 3; Table 2B).

#### Others

For two other non-steroidal nuclear receptors Rev-erb α/β (NR1D1/2) and RORα (NR1F1), data only from human patient biopsy is available. Whereas, *NR1D2* expression in UC patients is downregulated (72), *NR1F1* expression is upregulated in CD patients' colonic mucosa (208) (Table 2B).

### Orphan Nuclear Receptors

#### Hepatocyte Nuclear Factor 4 Alpha (HNF4α; NR2A1)

The orphan NR HNF4α is considered to be an important actor in intestinal epithelial cell homeostasis and mucosal barrier integrity as this NR regulates proper intestinal epithelial cell differentiation (209–211), lipid metabolism (212), goblet cell maturation, epithelial junctions and *Muc* gene expression (Table 3) (12, 211, 213, 214) (Figures 1, 2). Its role in liver and intestinal inflammatory networks has recently been reviewed in detail elsewhere (215). In humans, *HNF4A* expression is strongly reduced in intestinal biopsies of UC and CD patients (12, 64) and a GWAS has identified *HNF4A* locus as a susceptibility gene for UC (216). Besides, a single-nucleotide polymorphism within the *HNF4A* locus has also been associated with UC and pediatric CD (12, 216–219). Two HNF4α isoforms P1 and P2 are expressed in different compartments in the colonic epithelium, interact with distinct sets of proteins, and regulate the expression of unique sets of target genes, and thus play distinct roles during pathological conditions such as colitis (Table 3) (220). Pre-clinical mouse and human association studies suggest a highly important role for this NR, but as an orphan NR family member, currently, no agonistic compounds targeting HNF4α are available for treatment options.

#### Nuclear Receptor Subfamily 2 Group F Member 6 (NR2F6; EAR-2, COUP-TFIII)

We were the first to unravel the role of the COUP-TF family member NR2F6 in the pathogenesis of IBD (14). In immune cells, NR2F6 inhibits CD4^+^ Th17 T cell responses and autoimmunity (38, 221) arnd suppresses CD4^+^ and CD8^+^ T cell-driven anti-tumor immunity (40, 222, 223). In the gut, NR2F6 directly protects the colonic intestinal epithelium and thus enhances gut barrier homeostasis. *Nr2f6*-deficient mice are highly susceptible to DSS-induced colitis; mechanistically, NR2F6 directly binds to a consensus sequence at −2 kb of murine and human MUC2 promoter and transactivates Muc2 expression. Loss of NR2F6, therefore, increases intestinal permeability and results in spontaneous late-onset colitis in *Nr2f6*-deficient mice (14) (Figures 1, 2). Beside this pre-clinical dataset in mice, several studies from the literature document reduced *NR2F6* gene expression in patients with IBD (Table 3) (65, 72, 224, 225).

## Conclusion

NRs and NR ligands control important gastrointestinal functions ranging from nutrient uptake, the composition of the microbiota and intestinal immune cells. Mechanistic studies have identified several NRs involved in the pathophysiology of IBD; therefore, novel concepts integrating NR and gastrointestinal physiology have been integrated into the successful development of effective drug therapies into the clinic.

Despite this expanding use of NR targeting as a therapeutic approach, there are many unknown issues about some classes of NRs, especially orphan NRs. In addition to the translation of the existing knowledge on NR biology, advances in current knowledge especially assessing dynamic NR regulation throughout disease progression should lead to the development of new drug targets for treating IBD. In this context, it should be mentioned that currently NRs are investigated not only with a focus on gastrointestinal diseases but also from a broader perspective. NRs such as the GR (dexamethasone), RXR (bexarotene and alitretinoin), PPARα (fibrates), and PPARγ (thiazolidinediones) have already been successfully targeted by approved drugs for treating autoimmunity, cancer, hyperlipidemia, or type 2 diabetes, respectively. Understanding the molecular mechanism of NRs in other human diseases will hopefully provide important insights into how to optimize NR-targeting therapies in IBD.

## Author Contributions

VK and NH-K wrote the manuscript. ARM, HT, and GB contributed to the colitis and nuclear receptor biology aspects and helped with writing the manuscript.

### Conflict of Interest Statement

The authors declare that the research was conducted in the absence of any commercial or financial relationships that could be construed as a potential conflict of interest.
